# Plant Domestication and Resistance to Herbivory

**DOI:** 10.1155/2013/572784

**Published:** 2013-03-25

**Authors:** Bhupendra Chaudhary

**Affiliations:** School of Biotechnology, Gautam Buddha University, Greater Noida 201 308, India

## Abstract

Transformation of wild species into elite cultivars through “domestication” entails evolutionary responses in which plant populations adapt to selection. Domestication is a process characterized by the occurrence of key mutations in morphological, phenological, or utility genes, which leads to the increased adaptation and use of the plant; however, this process followed by modern plant breeding practices has presumably narrowed the genetic diversity in crop plants. The reduction of genetic diversity could result in “broad susceptibility” to newly emerging herbivores and pathogens, thereby threatening long-term crop retention. Different QTLs influencing herbivore resistance have also been identified, which overlap with other genes of small effect regulating resistance indicating the presence of pleiotropism or linkage between such genes. However, this reduction in genetic variability could be remunerated by introgression of novel traits from wild perhaps with antifeedant and antinutritional toxic components. Thus it is strongly believed that transgenic technologies may provide a radical and promising solution to combat herbivory as these avoid linkage drag and also the antifeedant angle. Here, important questions related to the temporal dynamics of resistance to herbivory and intricate genetic phenomenon with their impact on crop evolution are addressed and at times hypothesized for future validation.

## 1. Introduction

During speciation in crop plants, many morphological changes evolved in response to continuous selection pressure. Such characters are largely governed by genetic and epigenetic changes or are modulated according to ecological adaptations. The transition of wild progenitor species into modern elite cultivars through “domestication” entails evolutionary responses in which plant populations adapt to human selection. In response to this selection most plant species exhibit marked changes in a variety of phenotypes, most noticeably in traits consciously under selection (e.g., fruit size, yield, and evenness of maturation) [1]. As Darwin [2] profoundly recognized long ago, the study of the phenotypic variation between wild and domesticated plants presents an opportunity to generate insight into general principles of evolution, using the morphologically variable antecedent and descendant taxa. 

**Figure 1:**
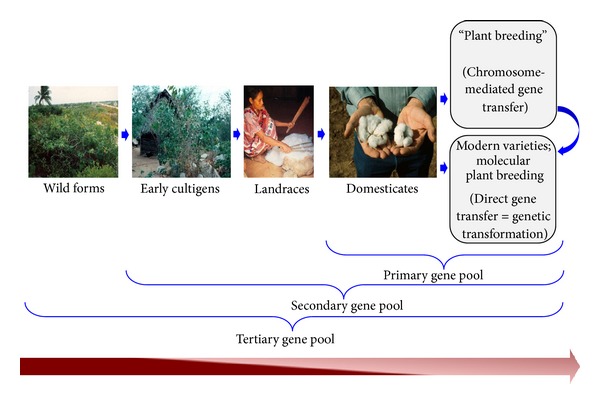
An example of cotton (*Gossypium*) evolution under human selection and contemporary breeding programs. The modern “crop” plants are the outcome of recurrent selection on wild form undergoing through early cultigens and landraces. In conventional and molecular breeding programs, it is possible to distinguish between primary, secondary, and tertiary gene pools and exchange of hereditary material. Each primary gene pool comprises one domesticated species together with those species with which it readily cross-breeds. The secondary gene pool includes species that can be cross-bred only with difficulty. The tertiary gene pool comprises those species which can be cross-bred only by using advanced techniques such as embryo rescue. (Courtesy Jonathan F. Wendel, ISU). The horizontal bar shows the reduction in genetic diversity along with domestication steps with the help of dark to lighter shades.

An example of how this concept has transformed our understanding is the realization that natural selection pressure, as well as adaptation under human selection, often led to unexpected and unexplained departures from predicted phenotypes. This mainly includes traits such as enhanced yield, enhanced apical dominance, reduced seed dormancy, perennial to annual habit, and relative susceptibility to pathogens, disease, and insect pests [3, 4]. However, the latter received the least attention during the process of “agricultural evolution.” The term “agricultural evolution” here, in fact, summarizes all of the changes accumulated in any wild plant form under natural selection, human-mediated artificial selection (=domestication), and modern breeding practices (Figure 1). From an evolutionary standpoint, these phenomena may be viewed as novel generators of variation in the tertiary gene pool comprised of domesticated and wild germplasms (Figure 1). Such variations occurred mostly at genetic level and provide the ability for a given species to evolve in response to the changing environmental conditions and stress factors [5, 6]. Notwithstanding the striking discoveries of the genetic basis of evolved morphological traits in crop plants [7–11], relatively little is understood about the manner in which gene networks and biological processes are associated with the more susceptible phenotypes of the elite forms.

Regardless of these rapidly accumulating insights, important questions remain about every stage of agricultural development. How did individual crop plants evolve from wild species and acquire agriculturally important traits? How do contemporary plant forms achieve diverse evolutionary trajectories separate from those of their progenitor(s)? How do recently formed elite accessions of crop plants become compromised of different resistance traits? To what degree has crop evolution via the process of domestication and concurrent breeding practices provided a stimulus for sustainable agriculture? Despite the domestication events followed by breeding practices across plant taxa, we do not know why agricultural evolution is so prevalent or conversely, why crop evolution is not universal if it confers some adaptive advantages and promotes species diversification. Nor do we understand the dynamics that underlie the transformation of wild forms to domesticated forms in cryptic crops such as cotton and corn.

It has been assumed that several agriculturally important traits such as resistance to abiotic and biotic stress conditions decreased significantly during evolution. For example, in domesticated accessions of the genus *Cajanus*, reduced levels of resistance have been reported against herbivores [12, 13], bacterial blight [14], and fungal diseases [15]. Among stress conditions, a reduction in drought tolerance, resistance to herbivory and pathogens, is the major threat to crop plants. It is difficult to understand what are the precise genetic underpinnings are that make a plant species vulnerable to drought, herbivores, and pathogens after passing through the evolutionary important mechanism of crop domestication? Surprisingly, what is the extent of reduction in resistance traits across crop plants, if the reduction in any particular resistance trait is proportional to another resistance trait? The answer to these questions may not be consistent across plant taxa but may only hold true for a particular plant lineage. Generally, domestication promotes heterozygosity leading to the more successful variants under selection pressure either through fixed hybridity or by polysomic inheritance. Could it be assumed that domesticated accessions are *in general* more “successful” than their wild progenitors? This is an exceedingly difficult question to answer, in large part because “success” is an ill-defined term that can refer to anything from short-term proliferation of individuals to long-term effects on lineage diversification. The susceptible nature of modern crop plant varieties in comparison to their wild progenitors could be one of the most apparent consequences of such a megaevent. Or accelerated mutational activity in coresident genomes (in case of polyploid crops) in early generations led to a downgrade in the pathogenic and herbivore resistance of domesticated plant species. Answering these and other questions will require comparisons of wild and domesticated forms by researchers from diverse disciplines such as ecology, population biology, and physiology. Unfortunately, these important areas of biology have received far less attention than the genetics and genomics of selection [7, 16–18]. Nevertheless, even in these better-studied areas much remains to be learned, and it is only by moving beyond the idiosyncrasies of a handful of model crop systems that emergent properties of selection can be detected. Though crop plants are threatened by many stress conditions, most of the previous research has been focused on the study of genetic end of resistance to herbivory at the gene expression level, during the process of selection, and at the phenotypic level. The aim of this paper is to provide a broad, updated entry to the literature as well as to highlight the major unanswered questions in the field of crop evolutionary genetics.

## 2. Evolution of Crop Plants

**Figure 2:**
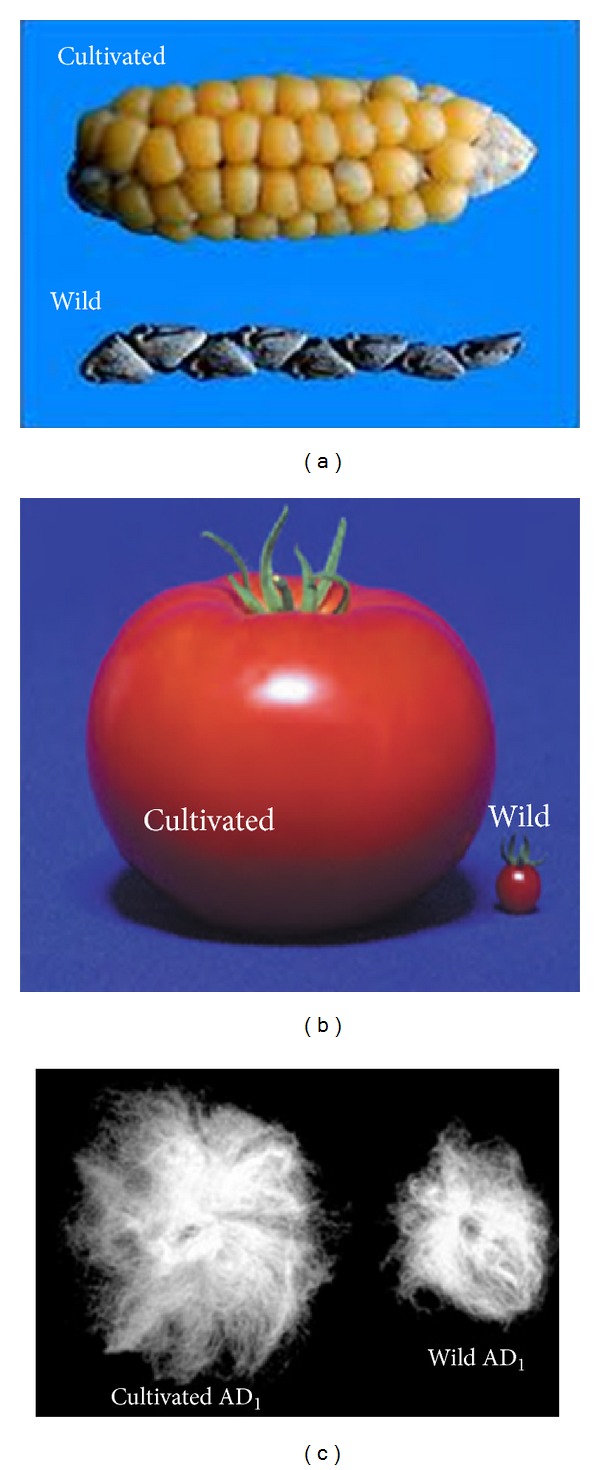
Difference in wild forms and their respective domesticated forms showing the impact of natural selection during evolution and domestication on phenotypic traits. (a) Evolution of maize; the domesticated maize (up) and wild teosinte (down). Teosinte has many lateral branches, while today's maize is unbranched. (b) Evolution of tomato; the much larger fruit (right) is from the domesticated *Solanum lycopersicum, *and the small tomato fruit (right) is from wild species *Solanum pimpinellifolium*; and (c) evolution of cotton (*Gossypium hirsutum*); the long fiber phenotype is from domesticated cotton *G. hirsutum* (AD_1_) (left) and small fuzzy phenotype is from the wild species (right).

A primary concern of agricultural evolution biology is to investigate where, when, and how crop plants originated. Vavilov's center of domestication [19] has been a valuable hypothesis as to where crops originated and where our sessile, agrarian cultures began. Since then, we have made great strides in pinpointing where contemporary domesticated forms have arisen and from which wild species they are derived (Figure 2). It has been believed that modern elite plant varieties with useful characters (mostly hybrids) for sustainable agriculture have been developed through (i) domestication and (ii) following research and breeding activities that were implemented by scientists and breeders worldwide (Table 1) (reviewed by [20, 21]). However, it is essential to understand which has been the foremost drive largely in the phenomenon of “agricultural evolution.”

The term “domestication” is often used to describe the process by which wild becomes stabilized. From the standpoint of morphological transformation, domesticated forms are by definition wild species with certain traits highlighted under human selection (Figure 1), showing character modifications including novel trait formation and subsequent segregation, for example, a reduction in grain shattering and seed dormancy in rice [10, 22, 23]; an increase in seed and pod size in *Vigna* [24]; an increase in fiber length and quality in cotton [16, 25]; changes in fruit size and shape in tomato [26]; increased apical dominance in maize [7, 8]; and more. As Darwin recognized [2], the study of the phenotypic variation between wild and domesticated plants presents an opportunity to generate insight into general principles of evolution, using morphologically variable antecedent and descendant in a comparative framework. This approach provides an intriguing perspective on the molecular genetics of human-mediated artificial selection. It is thus assumed that strong artificial selection coupled with introgression (=crossing with the respective wild relative) could drive the fixation of the most beneficial genes and their expression regulation in the process of crop domestication.

The domestication process and introgression under modern breeding programs must have served as effective means to increase the genetic diversity of elite cultivars especially following the initial domestication bottleneck, and to produce cultivars adaptive to climatic conditions [36]. But is it true for all domesticated crop plants? Can it be assumed that cereals with divergent genomic backgrounds experienced one or more domestication events in their evolutionary history? To test this hypothesis, metagenome studies of population genetic structures of cereals, other important modern taxa and their wild progenitors of known origin, are required. Instances of multiple independent domestications in cereals do not provide evidence for their clear ancestry (e.g., *Oryza, Hordeum*), and even if their respective wild progenitors were identified, the multiple origins of domesticated forms and their formation would mostly remain unknown. It has also been assumed that almost all crop plants have experienced repeated polyploidization of genomes in their evolutionary history with multiple-fold duplication of ancestral angiosperm (flowering plant) genes [37]. Polyploidy has influenced flowering plant diversification and provides raw material for the evolution of novelty by relaxing purifying selection on duplicated genes. However, following polyploidization, is the formation of multiple and independent domesticated forms a synchronized event across taxa? It still remains unclear whether genetic polyploids such as wheat, unlike rice and barley, must necessarily undergo mutation in orthologous loci simultaneously [38, 39]. In fact, many domesticated plant forms appear to undergo one or more mutations in a single gene, and thus the probability of parallel and independent selection of orthologous chromosomal regions responsible for the key domestication transition is expected to be weak.

Domestication could, therefore, include all responses to plant evolution, including genetic and epigenetic effects, as reviewed recently [1, 26]. What is the postdomestication impact on the genomic architecture of a plant? The domesticated forms are expected to undergo many of the genome-level megachanges both at structural and functional levels, such as sequence loss, structural rearrangements, and changes in the regulatory sequences, respectively [40, 41]. Recent large-scale microarray studies on the comparison of wild and domesticated forms of selective plant species confirmed that global gene expression had been radically altered by domestication [16, 25]. Such changes have certainly played an important role at the evolutionary scale as domesticated plant forms are not always achieved immediately, even in relatively simpler genomes, as reviewed by [17, 36]; and inheritable changes can only occur often through mutations at genic and/or regulatory levels. For example, a QTL (*sh4*) is responsible for the reduction of grain shattering in the wild rice [10] and a loss of function mutation of Vrs1 in six-rowed barley [9]; and a QTL is responsible for free-thrashing character in wheat [27]. Accordingly, what is the preference for such target genes to undergo mutations during selection process? It is now evident that the genes involved in important domestication transitions are preferably regulatory sequences whose mutations can generate substantial phenotypic modifications serving as suitable targets for strong artificial selection in the key steps of crop evolution [42, 43].

Regarding the magnitude of the changes that occurred throughout crop formation, it appears that breeding activities of crop plants are relatively of less radical change than the conversion of wild forms to the domesticated forms. If so, will the modern elite varieties derived through hybridization of two similar domesticated varieties/forms have less evolutionary resolution than those derived through hybridization with their wild progenitor species? This is definitely an affirmative assertion and has also been validated across plant taxa with reduced genetic diversity in the breeding programs. Thus, what may be the potential risks involved with such radical loss in the genetic diversity among elite cultivars? It may also develop the weedy competitors of crop plants as well as their susceptibility to the diseases, pathogens, and herbivores leading to severe crop losses. But is there any correlation between operative stress condition and the niche of any plant population? Are there sufficient pieces of evidence for relatively less epidemic of any particular pathogen or pest population? The bewildering possibility of such prevalence may destabilize the crop productivity as well as subsequent evolution. For example, despite low crop diversity among cereals initially, successful introgression of resistance to abiotic stress conditions, pathogens, and herbivores was deployed to maintain yield. In this scenario, what is the deadline for such incorporation of resistance traits in crops for sustainable agriculture? The answer is still unclear but it may only be possible if (i) domestication, (ii) change from traditional landraces to modern breeding varieties, and (iii) their over and above decade field adaptation can work indefinitely; for example, maize hybrids in the United States now have a useful lifetime of about 4 years, half of what it was 30 years ago [44].

The advent of genomics has brought a bonafide improvement to the study of such regulatory regions and generation of molecular and expression data, knowledge, and tools which could be applied in modern breeding programs for exploitation of genes from tertiary gene pool (Figure 1). To further understand the genetic basis of domestication, tremendous variations have been revealed using molecular markers. For example, in tomato, the genetic variation present in wild species has been investigated intensively for specific traits and is being exploited for tomato breeding [45–48]. Using DNA technologies, the diversity of domesticated tomato is estimated to comprise <5% of the genetic variation as compared to the rich reservoir in wild relatives [49]. So far, even using sensitive molecular markers, very low polymorphism within the domesticated tomato gene pool has been identified, [50, 51]. A loss of genetic diversity revealed through sequence repeat markers was also observed from wild *Triticum tauschii* to the landraces and subsequently to the elite germplasm [40]. Though the successful application of breeding programs has produced high-yielding crop varieties, ironically the plant breeding processes have threatened the genetic basis upon which the breeding depends. If assumed so, is the redundancy in the loss of diversity across taxa deleterious, or neutral? What is the spectrum of consequences of having genes and mutations underlying domestication transitions (colloquially called “domestication syndrome”)? Are the answers to these questions consistent among plant lineages or between parallel domestication events? Nevertheless, intensive artificial selection does not inevitably lead to a loss of genetic diversity, and diversity can be compensated by the introgression of novel germplasm. One example of this is the development of novel rice variety [10], with the introgression of major QTL, *sh4*, responsible for the reduction of grain shattering.

## 3. Resistance to Herbivory

### 3.1. Resistance Variations Arising by Domestication

Herbivores (insect pests) are the major factor responsible for destabilizing crop productivity in agricultural ecosystems. Herbivores have been recognized as a major constraint to crop production causing significant yield loss and quality degradation. In response to herbivory, plants have acquired inherent resistance against such pests; however, the intensity of this resistance varies enormously between wild and domesticated forms. So, what could be the rationale for such large variations in the resistance levels? It is believed that the alterations in herbivory resistance are a prominent outcome of genetic reduction within and between crop species. Several studies have shown that massive expression changes accompany crop evolution (under domestication and breeding practices). The magnitude of expression changes varied greatly between species influencing genetic diversity and resistance levels tremendously. However, do enough data exist at present to reveal general trends of reduced resistance to herbivory among domesticated forms across taxa? It is evident in three wild relatives of chickpeas that are, *Cicer microphyllum*, *C. canariense, *and *C. macracanthus *exhibiting significant reduction in leaf feeding, larval survival, and larval weight of neonate larvae of *Helicoverpa armigera *in comparison to domesticated perennial accessions. The extent of suppression of damage is impressive in wild accessions as compared to the domesticated chickpeas. 

Also, a comparison of global gene expression profiles in the wild and domesticated allotetraploid cotton *Gossypium barbadense* has specifically addressed the transcriptional effects of domestication during development. Several important genes and their functional categories related to resistance genes have been identified as downregulated in the domesticated form than its counterpart wild form supporting the assumption of reduced resistance properties of a plant species [16]. Furthermore, a comparison of wild and domesticated accessions of allopolyploid *G. barbadense* with their diploid progenitors (*G. arboreum *and *G. raimondii*; B. Chaudhary & J. F. Wendel, unpublished) revealed significant transcriptional downregulation of resistance-related genes in the expression phenotype of the domesticated form. This is a clear indication of reduction in herbivore resistance traits during the domestication of an allopolyploid crop. Since cotton allopolyploid species carry “A” and “D” genomes, derived from their diploid progenitors [52], it may also be argued that such a reduction in herbivory resistance may be the cumulative reduction occurred during both polyploid formation and domestication. However, following polyploid formation, many duplicated genes undergo transcriptional biases and do not behave as simple additive combinations of the parental genomes [53], but instead are maintained at the ancestral resistance levels at least in the wild forms. Thus, the possibility of reduction in resistance levels during polyploid formation appears to be minimal and major reductions in resistance levels may have occurred during the domestication process.

This lack of additivity in gene expression levels in most polyploid crop plants raises several fundamental questions on the consequences of the evolutionary dynamics of resistance gene expression following domestication. From a mechanistic standpoint, what is responsible for nonadditivity in gene expression, and why does this vary so much among resistance genes and between different genomic combinations? Why do such important genes from two diploid genomes demonstrate such a large disparity in the degree of suppression of the gene expression phenotype? Also from an evolutionary point of view, how does genomic evolution impact resistance gene expression variation during agricultural evolution (domestication followed by the breeding practices), and what are the potential phenotypic effects of each of these sources of variation?

### 3.2. Genetics of Resistance to Herbivory

One of the most recent and spectacular revelations in crop plants is the identification of a number of molecular markers, and how these markers could be applied to identify and track target genes in a marker-assisted breeding program [54–56]. Molecular markers have also been applied to increase understanding of the mechanistic and biochemical basis of herbivore resistance, as shown thoroughly in maize [57], mungbean [58], potato [59], and in soybean [60]. What are the putative regulatory genomic components and pathways providing resistance against herbivores, and at incredibly varied levels? Five QTLs have been identified in *Arabidopsis *known to regulate the glucosinolate-myrosinase system controlling the generalist herbivore *Trichoplusia ni *than specialist feeding insect *Plutella xylostella* [61] identified five QTLs in *Arabidopsis* known to regulate the glucosinolate-myrosinase system controlling the generalist herbivore *Trichoplusia ni* than specialist feeding insect *Plutella xylostella*. This demonstration of the higher levels of genetic variation for resistance to the generalist and specialist herbivores has been further verified and expanded in several subsequent studies, including one in which several QTLs from consistent resistance sources for leaf feeding insects SWCB and FAW were mapped on and observed to be located on chromosomes 6, 7, and 9 in corn. Given the resistance to both of these insects, candidate genes were identified as *mir *cysteine proteinase gene family [62] and the *Glossly15* gene controlling adult to juvenile transition [63]. Also in soybean, 81 QTLs related to herbivory resistance were identified through meta-analysis, and the locations of true QTLs were deduced with a confidence interval of 95% [64, 65]. Thus, could it be determined whether a genetic variant having a particular QTL or haplotype of a polymorphism is associated with the resistant traits? To understand this contention, a number of herbivory-resistance QTLs have been tested for nonrandom associations in the populations derived from a cross between resistant and susceptible parents determining their proportional contribution to the phenotype in wide array of crop species [59, 66]. RFLP-based identification of herbivory-resistance QTLs in maize revealed their strong association with antixenosis (=a resistance mechanism employed by a plant to deter or prevent pest colonisation) and antibiosis (=an association of two organisms in which one is harmed or killed by the other) resistance to corn earworm [57, 67], and also in soybean [60]. The herbivory resistance QTLs discovered by Rector et al. [67] accounted for most of the genotypic variance for corn earworm resistance in the susceptible *x* resistant hybrids; however, with some exceptions those could probably be addressed later with the help of soybean insect resistance QTL database. Will the contemporary catalogues of genome-sequencing projects across plant systems support the identification of important herbivory resistance loci? In result, it may definitely be assumed that future analyses based on whole-genome sequencing data will emphasize insect resistant (IR) QTLs/IR genes identified earlier through marker-assisted selection. This will substantially reduce the time utilized for their adaptive inheritance through classical or precision breeding. Until relatively recently, family-based QTL mapping and association mapping were the primary means of searching genes involved in crop evolution [68]. Considering these studies, different chromosomal regions were identified harboring corresponding QTLs involved in the herbivory resistance phenotype. Because there is a complex correlation among different cellular traits considered important for a resistant phenotype, it becomes enormously difficult to identify such specific biochemical constituents. This observation suggests that variation in resistance traits is controlled by intricate genetic mechanisms, a suggestion further bolstered by demonstrations of resistance variation in maize synthetic hybrids, whose genomes have not undergone any subsequent selection [7, 17, 69]. The mode of resistance is of great evolutionary interest, as it may often sporadically disappear under domestication and following breeding practices [7, 17, 69]. So, what are such vital target genes, their chromosomal positions, and putative structural changes those cumulatively have influenced the loss of resistance potential? Will any type of stochastic mutations in the coding or noncoding regions lead to the differential loss of resistance potential among elite cultivars? From an evolutionary perspective, it could be hypothesized that divergence in resistance potential at the genomic level may also preserve an extensive polymorphism, thus retaining additional raw material for subsequent evolutionary tinkering if exploited under breeding programmes.

## 4. Susceptibility to Herbivory and Acquired Resistance

### 4.1. Resistance Genes: Are There Patterns?

The domestication process increased a number of important traits required for agricultural innovations, though with few subsides. For example, the nascent “crop-form” (representative from independent domestication events) is more susceptible to herbivores as a result of having few important resistance loci, of which most were identified from their wild progenitor species. Is such phenotypic transformation under domestication universally advantageous or has accompanied with the loss of an “additional” benefit? What is the spectrum of consequences of having a set of important genomic loci selected under human selection? Are the answers to these questions consistent among plant lineages and/or between independent and parallel domestication events within a single species?

Traditional views maintain that domestication followed by breeding promoted the fixation of resistance loci, referred to as fixed heterozygosity [70–72]. It was thus suggested that inherited heterozygosity at resistance loci is beneficial. Modern views also support that introgression of resistance loci can be advantageous and provide a primary source of genes/alleles with new functions [73–75]. However, the identification of novel germplasm from the tertiary gene-pool is an enormously difficult task and it takes time for characterization. However, an inventive alternative is to carry out comprehensive genomic exploration of improved cultivars, primitive domesticated forms, and their wild progenitor species for the identification of candidate genes underlying resistance traits that show evidence of selection during domestication. With the latter approach, could the relationship between identified candidate genes with their phenotypic effects be envisaged? What are the confidence limits to forbid the possibility of the identified genes as false positives? The use of multiple statistical tests can certainly reduce such misreadings [76].

Analysis of a large number of loci underlying resistance to herbivory in soybean showed that resistance is an outcome of a mixture of major and minor gene effects and is not random. Some loci responsible for acquiring resistance to herbivory were underlying within regions having loci for the resistance against cyst nematode [77]. Classification of different soybean genotypes showing broad resistance has suggested important loci contributing to active synthesis and accumulation of products to stop (=antixenosis), deter (=antibiosis), and/or administer (for which mechanism is not readily established) herbivory [55, 67]. Three QTLs influencing resistance to corn borer species in maize have also been identified as overlapping with other genes of small effect in regulating the resistance phenotype, indicating the presence of pleiotropism or linkage between genes affecting resistance and other agronomic traits [78]. Recently, one major and three minor QTLs in rice have been identified as showing resistance against green rice leafhopper along with defined microsatellites for marker-assisted selection [79]. However, at present, relatively little is understood about the temporal dynamics of resistance to herbivory in different crop plants, and this requires the study of multiple genomes with the empirical reality of long-term resistance to herbivory.

### 4.2. Modern Cultivars Are More Susceptible Than Their Wild Progenitor Species

Under classical models, plant resistance to herbivory and pathogens is proposed to be a primary phenotype mostly available in the wild ancestors. For instance, plant introductions (PIs) in soybean with low agronomic quality have been demonstrated to be resistant against number of defoliating insects [12, 13]. Such models agree with the theory predicting that domestication occurred by human-mediated exertion through artificial selection on a wild species, both positive and negative, over hundreds of generations resulting in the development of cultivable species. In general, wild plant forms resist attack by herbivores and pathogens mainly through constitutive and inducible defense mechanisms [80]. The evolution and maintenance of the latter are now firmly accepted as an integral component of the plant defense mechanism against herbivores. However, the question remains when, where, and how induced resistance is deployed? Based on differences in the signaling pathways and spectra of effectiveness, the induced resistance could be categorized into (i) systemic acquired resistance (SAR) occurring in the distal plant parts following localized infections and (ii) induced systemic resistance (ISR) stimulated by nonpathogenic organisms and is regulated by jasmonic acid and ethylene [81]. During crop evolution (domestication followed by breeding practices), besides all evolutionarily relevant “internal” costs (genetic or allocation) of induced resistance, what are the other costs that may also be influencing the resistance phenotypes? There has been rapid progress in the detection of other important components, such as ecological costs, which are the result of a plant's interaction with its environment. Therefore, the conceptual separation of genetic and environmental contributions throughout crop formation would help in our understanding of induced resistance [82, 83]. 

If artificial selection prevails all through generations at the genomic level, is the operation of such selection global or localized? It may be argued that the evolutionary event such as domestication can affect the sequence variation at wide-reaching loci within a crop plant. In that scenario, what could be the limiting factors responsible for such proposed “genetic erosion” in an elite germplasm? One explanation could be that selection in modern breeding programs instead acts on selected important loci controlling a variety of traits, concluding that selection in either case would significantly reduce species-wide polymorphism and make it more vulnerable to the stressful conditions [4]. Besides intensive selection in modern breeding programs, the narrow genetic base is often cited as a contributing factor to low diversity, at least in soybean. Analysis of 111 fragments from 102 genes in four soybean populations showed evidence of a reduction in genetic diversity within and around the selected loci creating genetic bottlenecks [3]. The reduction of genetic diversity at different loci could result in “broad susceptibility” to newly emerging diseases and herbivores, thereby threatening long-term food and feed security [4]. One or multiple domestication events in the evolutionary history of soybean provide a discernible degree of diversity compensation that is up to a 50% reduction, eliminating almost 81% rare alleles present in the wild soybean (*G. soja*), and even appear to undergo significant change in the allele frequency [3]. Could it be assumed that the most significant loss of diversity in modern cultivars occurred during domestication, or due to an unusually low level of initial genetic variability in the wild progenitor, or both? It seems that the major loss of diversity occurred during domestication leading to the bottleneck where there was a loss of rare alleles present in the wild form and landraces. Contrary to classical predictions that loci under selection pressure may be relatively free to acquire heritable changes, it has been shown among subspecies of maize and rice that single nucleotide polymorphisms encode radical changes in the regulatory regions preferentially preserved in the domesticated forms and evolve conservatively [7, 17, 57]. Any such change can lead to divergence in subgenomic expression components, for example, in allopolyploid crop plants [53]. Those in result may influence the quantitatively inherited traits such as resistance to herbivory. Since wild plant forms have large genetic diversity that could be exploited for introgression of important traits in the modern varieties, it may be assumed that some sources of resistance have been left behind during plant domestication. However, it would be relatively difficult or even unsuccessful to introgress such traits into modern cultivars because this may increase the potential of inferior yield through linkage drag as also shown earlier [84, 85]. Moreover, it will also be difficult because crops with antifeedants from wild may have toxicity and anti-nutritional angle. Here the author has a strong opinion that transgenic technologies may provide a radical solution to the herbivory as these avoid linkage drag and also the antifeedant angle.

## 5. Transgenic Resistance Mediated by the Expression of Foreign Proteins

### 5.1. Transgenic Crops and Resistance to Herbivory

Introduction of novel foreign genes into crop plants helps breeders to extend their germplasm with novel phenotypic traits. Such required traits are often related to the control of abiotic and biotic stresses, increasing the crop yield and improving the product quality, which were hitherto difficult or not possible to breed using a conventional approach [86, 87]. Given the toxicity of chemical pesticides, for the past two decades, a major emphasis has been on the control of herbivory through more rational strategies such as Integrated Pest Management (IPM). A component of IPM is the use of naturally available pesticides such as plant secondary metabolites and expression of heterologous proteins. Transgenic crops with a “modified” single gene developed for herbivore resistance are immensely beneficial in economic, environmental, and health concerns, as recently reviewed [88, 89], and understood to be “second generation” resistant crops (detailed in next section). A number of genes have been discovered which are toxic or antifeedant to herbivores and could be of plant or bacterial origin. A major contribution of herbivory-proof crops is in the reduction in application of harmful insecticides sprays and subsequent increase in crop yield [90, 91]. For example, transgenic maize event MON863 developed using a wild type gene from bacterium *Bacillus thuringiensis* (*Bt*), resistant to corn rootworm, was first commercialized in the USA in 2003 [92] and successfully grown until recently. In such a scenario, how do we provide a global perspective of the status of biotech crops? The easiest way is to calculate the global adoption rates as a percentage of the global areas of principal crops (i.e., soybean, cotton, maize, and canola) in which biotechnology is utilized. Though, during last one decade, herbicide tolerance has consistently been the dominant trait, deployment of multiple genes for other traits such as resistance to herbivores is becoming increasingly important and most prevalent for sustainable agriculture. The best example of the dynamics of this very rapid adoption is the contemporary biotech maize for stacked traits [93]. However, at the field level evaluation, the best-studied resistance to the herbivory phenomenon in the crops is the *Bt* cotton [94], which has a documented reduction in pesticide application of more than 70% in the developing countries, utmost domesticated in India [91], though results have been very variable. In 2009, 5.6 million small and marginal resource farmers planted and benefited from ~8.4 mHa of *Bt* cotton, equivalent to 8.7% of the total area under cotton cultivation in India. The corresponding adoption rate of biotech cotton has also been increased globally from 15.5 mHa to 16.1 mHa in the year 2009 [93]. For soybean, the global hectarage of herbicide tolerant transgenics was 69.2 mHa, (up by 3.4 mHa in 2009), which leads this crop to be the largest GM crop grown worldwide [93]. These examples illustrate two major contributions of the biotech approach to plant breeding: (i) enlarging the gene pool by including novel genes that breeders could not access by crossing techniques and (ii) modifying the genes by recombinant DNA technologies to fine-tune transgene expression [95]. The latter needs more emphasis and attention to achieve success in transgenic technologies for improved traits in the crop plants.

What controls the level of a foreign gene expression in genetically modified plants? Any or all of the molecular mechanisms associated with cellular gene expression machinery could be involved. Such a definition encompasses an array of molecular mechanisms at the transcriptional level including DNA methylation, mRNA decay, and small RNA-mediated gene silencing or at translational level having protein misfolding, degradation or other modification, and nuclear/chromosomal context with respect to genomic location of transgene often referred to as “position effect.” It is generally assumed that because genes of prokaryotic origin are expressed poorly in higher organisms, such as plants [96, 97], certain modifications in such genes are required in order to achieve optimal expression. This may include modification in the GC content (as plants are comparatively GC rich than bacteria particularly *Bacillus* spp.), and in codon usage [97], after the introduction of regulatory sequences and polyadenylation signals. This fact is well supported with a significant increase in the expression of a codon-modified *Bt cry1Ac* gene in comparison to the wild-type gene from bacterial origin. Subsequently, by modifying codon usage, removing polyadenylation sequences [96], and other modifications in *Bt* genes, a number of plants have been transformed against their target pests [98, 99]. But is this true only for bacterial genes? Can it be assumed that plant-derived genes with insecticidal potential [100, 101] such as protease-inhibitors, alpha-amylase inhibitor, lectins, and hemilectins do not require any modification for their optimal efficiency prior to the delivery into other crop systems?

Under field conditions, reverse effects of transgenics were also recorded with (i) harmful effect on nontarget insects and (ii) target insect to develop resistance against insecticidal genes used. The first risk could be addressed by designing the synthetic genes to target the hypervariable regions of target insect genes, thereby avoiding their lethal effects on nontarget insect population. However, in the latter case, the potential hazard could only be equilibrated through achieving very high transgene expression in the transgenics through the system-specific codon usage modification of a gene, use of high strength constitutive promoters, “position effect-” based screening of large transgenic population, and following refuge strategy to delay the acquired resistance in the pest population. If transgenic technologies are so promising and successful over conventional breeding, what are the major constraints that delayed the worldwide cultivation of genetically modified (GM) crops? Here, one of the likely explanations may be the inappropriate resistance management strategies deployed so far for the commercially important crop plants. For rice, assessment of agricultural fields for productivity and health effects in China emphasized such issues and highlighted key concerns on policy implementation and resolution of trade barriers [102], which may also be true globally. Clearly, much remains to be learned about such issues, as how should GM crops for herbivory resistance be synchronized for sustainable agriculture?

### 5.2. Next Generation Herbivory-Resistant Crop Plants

Transgenic technologies are undoubtedly important for plant defense against stresses, and it has also been argued that they are useful for the incorporation of novel phenotypes into crop plants. Existing single gene biotech crop studies may be extrapolated to a hypothetical case where full coverage of all target herbivores and plant diseases would be available in a genetic stock. As mentioned previously, biotech maize in USA is the best example of the deployment of stacked multiple traits including *Bt* genes (one to control the European corn borer complex and the other to control rootworm) and herbicide tolerance (first commercialized in 2005) and continued to grow in 2009 [93]. However, hints regarding gene pyramiding, exclusively for herbivory resistance, have also been emerging rapidly in last decade. In rice (*Oryza indica*), incorporation of two *Bt* genes and one lectin gene showed the control of three major herbivores: rice leaf folder (*Cnaphalocrocis medinalis*), yellow stemborer (*Scirpophaga incertulas*), and the brown planthopper (*Nilaparvata lugens*), respectively [103]. This indicates that, in rice, the long-term effect of multiple gene expression is an apparent enhancement of the resistance phenotype established by the synergistic effects of transgenes. Also, two *Bt* gene-transgenic cotton showed enhanced protection against *Helicoverpa zea* in comparison to the single gene transgenics with any of the two genes studied [104]. Evidence from our research laboratory on cotton indicates that transgenic stock with two *Bt* genes targeting two different lepidopteran insects shows significant improvement in the resistance trait against individual insects than do the respective single gene transgenics (B. Chaudhary and D. Pental, unpublished data). This indicates that the gene combinations may have played a strong role in reserving a durable resistance phenotype. Even without knowing the comprehensive specific mechanism(s) involved, there is clearly some association between the observation of high levels of protection against herbivores and the presence of multiple genes.

However, for any particular trait such as resistance to herbivores, can only genes from similar origin be tagged for synergistic activity? Can it be assumed that genes from distant origin be grouped together for increased resistance phenotype? The answer lies with the gut anatomy of phytophagous herbivore, which has different binding sites for toxic proteins that determine the spectrum of different lethal protein(s) activity and severity and provide clues for the use of diverse toxic genes to introgress herbivory resistance phenotype. The use of lectins, for example, *Galanthus nivalis* agglutinin (GNA), with *Bt* genes is very promising for increased insect resistance, with the ability of GNA to serve as carrier protein for the delivery of insecticidal proteins (in most cases *Bt* toxins)[105].

What should be the major selection criteria for insecticidal proteins when used for gene pyramiding? This may entail (i) toxic activity against wide spectrum of herbivores and (ii) nonhomology between or among concurrent toxins used. A well-studied example in second-generation insect-resistant transgenic plants is the use of novel vegetative insecticidal proteins (VIPs), which are produced by *Bacillus thuringiensis* during its vegetative growth along with *Bt* crystal proteins. Unlike *Bt* crystal proteins known as *δ*-endotoxins, VIPs are not parasporal and are secreted from the bacterial cell during vegetative growth. The full-length toxin gets activated proteolytically to a core toxin by proteases in the lepidopteran gut juice [106, 107]. Since the mode of action, structure, and binding sites of VIPs are different from *Bt* toxins in the insect gut epithelium, their use as potential insecticidal proteins for gene staking is very promising. Transgenic cotton expressing two insecticidal proteins Vip3A and Cry1Ab is estimated to be highly effective against two cotton herbivores, *Helicoverpa armigera* and *Heliothis virescens* [108]. Hence, it is assumed that enhanced resistance could be achieved by using two or more effective analogous insecticidal proteins. Gene combination *Bt cry1Ac* and snowdrop lectin GNA were also tested in cotton and showed resistance against insect pests *Heliothis armigera* and *Aphis gossypii* [109]. Also, the results from a comparison between single *Bt* toxin tobacco transgenics and two gene transgenics having the *Bt* gene and the cowpea trypsin inhibitor (CpTi) gene showed the dominance of two gene products with enhanced insecticidal efficacy against cotton bollworm (*Helicoverpa armigera*) [110]. Further, an evaluation of *Bt*-CpTi fusion protein was performed in *Brassica oleracea* to study the insecticidal effect of this fusion protein on cabbage worm, which showed high activity of trypsin inhibitor, and the overall strong resistance to the common cabbage worm [111]. The synergistic activity between unrelated genes seems very promising. Having distinct binding sites of different *Bt* genes in the mid-gut of herbivores, novel combinations of such genes have been considered good to be deployed in delaying the evolution of resistant herbivores [112]. Since most of the activated Bt toxins share a common three-domain structure with a similar mode of action [113, 114], it is possible to develop a hybrid toxin through domain swapping [115, 116]. Transgenic cotton and tobacco with a hybrid Cry1EC toxin developed against polyphagus insect *Spodoptera litura* resulted in extreme toxicity to all developmental stages of larval development [116]. Naimov et al. [117] constructed a hybrid *Bt* gene using truncated *cry1Ba* gene as a scaffold and inserted part of the second domain of *cryIIa* gene conferring resistance to both coleopteran and lepidopteran pests. These studies support the assumption that such a novel strategy may provide new avenues for resistance management studies involving multiple transgenes in crop plants [88].

The pyramiding technology has been noted to provide excellent control of a broad range of herbivores and reinforces the argument that developed resistance in selected herbivores against one toxin will still be fully susceptible to other toxin molecules present in the plants (reviewed by [118]). If so, is gene pyramiding an enduring strategy for sustainable resistance? Given the insecticidal activity, it appears very clear that single gene transgenics in the field cannot be sustained without an integrated approach [119]. Such approach will definitely delay or, with other components of IPM strategies, preclude the possible adaptation of herbivore-populations to resistant transgenic plants.

### 5.3. Endogenous Resistance to Herbivores is Prolonged by Transgene Stacking

As mentioned above, one strategy to delay the evolution of resistant herbivores is the stacking of multiple *Bt* genes [120, 121]. However, major concerns with this approach are (i) limited insecticidal properties of *Bt* genes to the target herbivores due to high specificity of Bt toxins, (ii) the potential cross-resistance leading to the evolution of resistant herbivores and (iii) the restricted use of possible novel combinations of *Bt* genes. A possible solution to the aforementioned problems may be either pyramiding of *Bt* genes with another transgene having a different mode of action, as also discussed earlier [108–110], or by pyramiding the native herbivory resistance genes with selected *Bt* transgenes (reviewed in [64]). In reference to the latter, Walker et al. [55] identified that when the SSR-based IR-QTL conditioning corn earworm resistance in soybean was combined with *Btcry1Ac* gene, it resulted in detrimental effects on the larval weights, and with the least foliage consumption. With these results, can it be assumed that the combined effects of endogenous resistance and transgene-based resistance are additive while having independent mode of action(s)? Genetic modification of cotton with the *Btcry1Ab* transgene with high terpenoid levels showed more resistance to tobacco budworm than transgenics with low terpenoid levels [122]. However, transgenics developed in susceptible potato with *Btcry3A* gene against Colorado potato beetle larvae exhibit higher or at least similar mortality in the target herbivores as in the resistant potato line with leptine glycoalkaloids [123]. In the latter scenario, a better strategy may be the identification of IR-QTLs with the help of molecular markers, rather than with specific traits or compounds known to be associated with the resistance phenotype, as was earlier exampled in soybean, cotton, and potato [55, 122, 123]. The native plant resistance is suggested to be advantageous also in the controlling of the resistant herbivore populations, as demonstrated in the case of tobacco budworm, providing the “lethal dose” required for resistance management strategy [124]. Thus, staking multiple *Bt *genes along with the exploitation of native resistance through marker-assisted breeding will comprise complementary additive effects ameliorating the deployment of resistance management practices in the field.

## 6. Conclusions

What are the key evolutionary attributes that make the conversion of wild germplasm to “crop” such a prevalent phenomenon? Are the most important evolutionary properties of modern crop plants due to contemporary breeding efforts *per se*, or is domestication, artificial selection, just as important? In the opinion of a crop breeder, the end product is warranted by both. Plant breeding of independently selected domesticated forms began almost ten thousand years ago. Gene mutations occurred during selection, polyploidy, or artificial or natural hybridization and brought remarkable genetic variations. For management of stress conditions in crops, do parallel breeding efforts share similar genomic modifications or is it system/event specific, given the likelihood that different features of human-mediated selection may predominate among various lineages? 

Very important successes during domestication in terms of crop yield and quality with other agronomic aspects have been achieved, but with compromised resistance to the herbivores and diseases. All modern crop plants are protected against herbivores by using synthetic toxic chemicals, as has been the case for many years, and this often leads to the development of resistance in herbivores against such frequently used chemicals. To circumvent this problem, introgression of important traits from the wild gene pool has been performed through classical breeding, but has met limited success due to incompatibility and numerous genetic and genomic differences among plant forms. Such principal distinctions may be critical, in that the interactions established by the initial conditions propagate from the time of initial origin through periods of stabilization and long-term evolutionary outcome.

Based on the limited number of available examples, when gene transfer occurred in the nascent interspecific crosses, genetic engineering techniques had proven useful to overcome such problems. Two potential benefits of genetic modification are precise transfer of foreign gene sequences and the evolution of adaptive transgressive traits. Two potential benefits of genetic modification are precise transfer of foreign gene sequences and the evolution of adaptive transgressive traits. Classic plant breeding programs are reinstated for heterozygosity and therefore may be more likely to experience locus-specific linkage with its evolutionary consequences. In contrast, a transgenic crop plant possesses a greater insertion of multiple alien genic sequences with immediate phenotypic effect and genetic novelty. Indeed, experimental analysis of genetically modified crops for multiple traits suggests that pyramiding of favorable genes for individual trait may yield a “super-crop” with high returns. Thus, the breadth of recurrently selected traits in the domesticated plants and the genetic transformation system together has a major effect on the creation and retention of evolutionary novelty in the crop system.

## Figures and Tables

**Table 1:** Some centers of origin of crop domestication and the trait under selection.

Crop	Area of origin	Traits influenced under domestication	Source
Cereals			
Rice	China	Reduction in grain shattering and seed dormancy; synchronization of seed maturation; reduction in tiller number; increase in tiller erectness; increase in panicle branches; Number of spikelets per panicle; reduction in hull and pericarp coloration and awn length	[10, 22, 23]
Barley	Fertile crescent, and Israel-Jordan area	Reduction in grain shattering; separation of seeds from hulls	[9]
Wheat	Southwest Asia (fertile crescent)	Reduction in shattering of grains (nonbrittle rachis); free-threshing trait	[27–29]
Maize	Mesoamerica	Increased apical dominance; production of seeds in relatively large numbers	[7, 8]

Brassicas			
Cabbage	—	Large number of leaves surrounding the terminal bud	[30]
Cauliflower	—	Formation of inflorescence meristems	[31]

Legumes			
Lentil	Mesoamerica	Seed dormancy	[32]
Vigna	Southeast Asia	Increase in seed and pod size, nontwining growth habit, loss of seed dormancy, and seed dispersal ability	[24]
Pea	Southwest Asia (fertile crescent)	Indehiscent pods; lack of dormancy dwarfness; less basal branches; large seeds; good seed quality day neutral flowering	[33]

Fibers			
Cotton	Mexico and Peru	Fiber length and quality	[16, 25]

Vegetables			
Tomato	Mesoamerica	Fruits' size, shape, and structure	[4, 26]
Potato	Andes and Amazonia	Shorter stolons, larger tubers, (often) colored and variously shaped tubers, and reduction of bitter tuber glycoalkaloids	[34]
Squash	Mesoamerica	increased seed length and peduncle diameter, change in fruit shape and color	[35]
